# Scenario-Driven Supply Chain Charaterization Using a Multi-Dimensional Approach

**DOI:** 10.1007/978-3-030-63505-3_4

**Published:** 2020-10-23

**Authors:** Ana Cristina Barros, Pedro Pinho Senna, Irene Marchiori, Dimitra Kalaitzi, Sébastien Balech

**Affiliations:** 2 STIIMA-CNR, Milano, Italy; 3 Fraunhofer IML, Dortmund, Nordrhein-Westfalen Germany; 4 INESC TEC, Porto, Portugal; 5 Aston University, Birmingham, UK; 6 INESC TEC - Institute for Systems and Computer Engineering, Technology and Science, Campus da FEUP, Rua Dr. Roberto Frias, 4200-465 Porto, Portugal; 7 STIIMA-CNR - Institute of Intelligent Industrial Technologies and Systems for Advanced Manufacturing, National Council of Research, Via Alfonso Corti, 12 20133 Milan, Italy; 8 Institute of Electronics, Computer and Telecommunication Engineering, National Council of Research (IEIIT-CNR), c/o Università di Padova, via Gradenigo 6/B, 35131 Padova, Italy; 9 Department of Engineering Systems & Supply Chain Management, College of Engineering and Physical Sciences, Aston University, Aston Triangle, B4 7ET Birmingham, UK; 10 PNO Consultants, Avenue de la Joyeuse Entrée 1, 1040 Brussels, Belgium

**Keywords:** Macro-scenarios for future supply chains, Supply chain configuration, Supply chain characterization

## Abstract

Extreme disruptive events, such as the volcano eruption in Iceland, the Japanese tsunami, and the COVID-19 pandemic, as well as constant changes in customers’ needs and expectations, have forced supply chains to continuously adapt to new environments. Consequently, it is paramount to understand the supply chain characteristics for possible future scenarios, in order to know how to respond to threats and take advantage of the opportunities that the next years will bring. This chapter focuses on describing the characteristics of the supply chain in each of the six macro-scenarios presented in Sardesai et al. (2020b), as final stage of the scenario building methodology. Supply chains for each scenario are characterized in eight dimensions: Products and Services, Supply Chain Paradigm, Sourcing and Distribution, Technology Level, Supply Chain Configuration, Manufacturing Systems, Sales Channel, and Sustainability.

## Introduction

The late 1990s is often described as the time when the term supply chain management started to gain popularity. While the advent of globalisation increased competition and allowed for the search of new products, higher quality and lower cost, customisation brought new challenges to suppliers in order to fit the costumers’ unique needs and expectations. Consequently, those years were characterised by massive changes in the way that companies interacted with each other and with customers (Min et al., 2019).

Since then, the ever-shifting global economy brings great challenges to companies and supply chains, as customers continually demand improvements in products and services and, at the same time, require lower prices, new sales channels, faster deliveries and social and environmental responsibility (Zimmermann et al., 2016). Looking into the future and trying to understand how to compare the possible scenarios and the corresponding innumerable challenges, as well as how to take advantage of the opportunities that the next years will certainly bring, are challenging tasks. These have to be faced by companies and governments in order to keep or increase competitiveness and to be able to create resilient and sustainable supply chain over time (Calatayud et al., 2019).

This chapter considers the macro-scenarios presented in Sardesai et al. (2020b) to discuss the fit of the supply chains, in terms of its overall characteristics. Thus, the research question driving this chapter is: *Which are the supply chain characteristics for the six macro*-*scenarios for Europe in 2030?*

This objective follows the scenario building method and describes the supply chains for each future macro-scenario based on the approach of the Consequence Matrix (Sardesai et al. 2020a). The idea is to provide insights into how the trends of each macro-scenarios can influence the behaviour of the companies and the supply chains. The results are a set of descriptions of the supply chains for the future macro-scenarios based on eight dimensions: Product and Service, Supply Chain Paradigm, Technology Level, Sourcing and Distribution, Supply Chain Configuration, Manufacturing Systems, Sales Channels and Sustainability.

## Methodology

The research method followed to define the characteristics of the supply chains for the macro-scenarios for Europe 2030 is based on the consequence analysis of the scenario projections for various decision fields and is presented in Sardesai et al. (2020a). This consequence analysis is then represented in the “Consequence Matrix”, which is the basis of the scenario transfer phase of Gausemeier et al. (1998) approach to scenario building (Sardesai et al. 2020a).

The decision fields for the Consequence Matrix were defined through literature review. As a first step, decision fields from the literature review were organized using the Product—Process—Supply Chain framework, also called three-dimensional concurrent engineering (Marsillac and Roh, 2014). Afterwards, the decision fields were re-organized into a final list of eight decision fields for the characterization of supply chains: Products and Services; Supply Chain Paradigm; Sourcing and Distribution; Technology Level; Supply Chain Configuration; Manufacturing Systems; Sales Channel; and Sustainability. Table 1 presents the list of the eight decision fields together with the alternatives for each of them.

**Table 1:** Decision fields for the consequence matrix

Decision field	Description	Alternative characteristics
Product and service (Fisher, 1997; Aitken, 2003; Godsell et al., 2011; Von Haartman, 2012)	Selection of the main type of products and services of the supply chains based on the demand characteristics of each macro-scenario.	**Mainstream Products**: standard, high volume markets **Customized Products**: personalized, high variety markets **Frugal Products**: low cost products with low maintenance and repair, while also providing robustness, user friendliness and economies of scale (Rosca et al., 2017) **Servitization:** transformation of products as stand-alone selling items towards package based customizable products with services attached (Vendrell-Herrero et al., 2017)
Supply chain paradigm (Lee, 2002; Wagner et al., 2012)	Selection of the supply chain paradigm based on the uncertainty level of demand and supply in each macro-scenario	**Efficient**: low uncertainty in demand and supply **Agile**: high uncertainty in demand and supply **Leagile**: high uncertainty in demand and low uncertainty in supply **Risk-hedging**: low uncertainty in demand and high uncertainty in supply
Sourcing and distribution (Christopher, 2016)	Selection of the widespread of sourcing and distribution in the supply chains for each macro-scenario	**Global**: sourcing/distribution is global **Local**: sourcing/distribution is local **Glocal**: mix between global and local sourcing/distribution
Technology level (Capgemini Consulting, 2013)	Selection of the digital mastery (i.e. technology-enabled initiatives regarding customer experience and internal operations) of supply chains in each macro-scenario	**Digital Masters**: strong adoption of digital technologies in the supply chain **Tech Fashionistas**: adoption of advanced digital technologies in segmented niches **Tech Beginners**: experimentation with technological adoption **Tech Conservatives**: strong presence of traditional technologies
Supply chain configuration (Gereffi and Lee, 2012)	Selection of the type of supply chain configuration for each macro-scenario, based on its governance, design and technology level	**Hyperconnected Factories**: integrated, network-based smart factories (Park, 2016) **Modular Systems**: decomposition of complex products and processes in modules that may be replicated in several supply chain echelons **Urban Manufacturing**: small-scale distributed production systems in cities (Kumar et al., 2016) **Simple Systems:** used for frugal mass products towards avoiding maintenance and repair (Rosca et al., 2017)
Manufacturing systems (da Silveira and Sousa, 2010)	Selection of the manufacturing strategy considering its fit with the demand characteristics and with the capabilities of supply chains in each macro-scenario	**Digital Lean Manufacturing**: Adding value by eliminating waste with a set of management practices and techniques supported by the implementation of digital technologies. Long-term relationship between manufacturer and supplier. (Arlbjorn and Freytag, 2013) **Digital Mass Customization**: Focused on broad provision of customized products and services by modularizing design, having flexible processes and allowing for integration (allowed by digital technologies) between supply chain members. Manufacturers provide affordable customization. (Fogliatto et al., 2012) **Agile Manufacturing**: Comprehensive response to business challenges of profiting from rapidly changing, continually fragmenting, global markets for high quality, high performance, customer configured goods and services. It is the ability to compete and prosper within a state of dynamic change. Aimed towards satisfying customers by configuring to order, it allows for unpredictability with strategies to face uncertainties. (Zhang and Sharifi, 2007) **Flexible Manufacturing**: Adaptation to customers preferences and changing need∀s. It must react with little penalty time, being either reactive (aimed at environmental uncertainty) or proactive (organization will redefine market uncertainties and influence customers desires). (Jain et al., 2013) **Efficient and Reconfigurable Manufacturing**: Classified in terms of the levels regarding decision-making and action-taking, where lower levels influence hardware changes and higher levels impact software changes or different choice of alternative methods/organization structures. It has the ability to reconfigure hardware and control resources at all functional and organizational levels, aimed at quickly adjusting production capacity and functionality as a response to sudden changes in market or in regulatory requirements. (Bi et al., 2008)
Sales channel (Beck and Rygl, 2015	Selection of the mechanism to sell products and services to customers in each macro-scenario	**Omnichannel:** integrated multichannel approach that delivers seamless customer experience across various online and offline channels (Hansen and Sia 2015) **Consumer to Consumer (C2C)**: consumers interact directly with each other to do business (Dan 2014) **Traditional Sales Channels**: products are sold through shops, stores and malls
Sustainability (Seuring and Müller, 2008)	Selection of the environmental and/or social focus of the supply chain in each macro-scenario	**Green supply chains**: focus on reducing environmental and ecological impacts (Batista et al., 2018) **Closed-loop supply chains**: focus on product returns (Guide and Van Wassenhove, 2006) **Resource-efficient supply chains**: focus on strategies to deal with resource scarcity (Matopoulos et al., 2015) **Social-responsible supply chains**: focus on social responsibility (Tang, 2018) **Humanitarian supply chains**: focus on coordination mechanisms in disaster relief (Balcik et al., 2010)

**Table 2:**
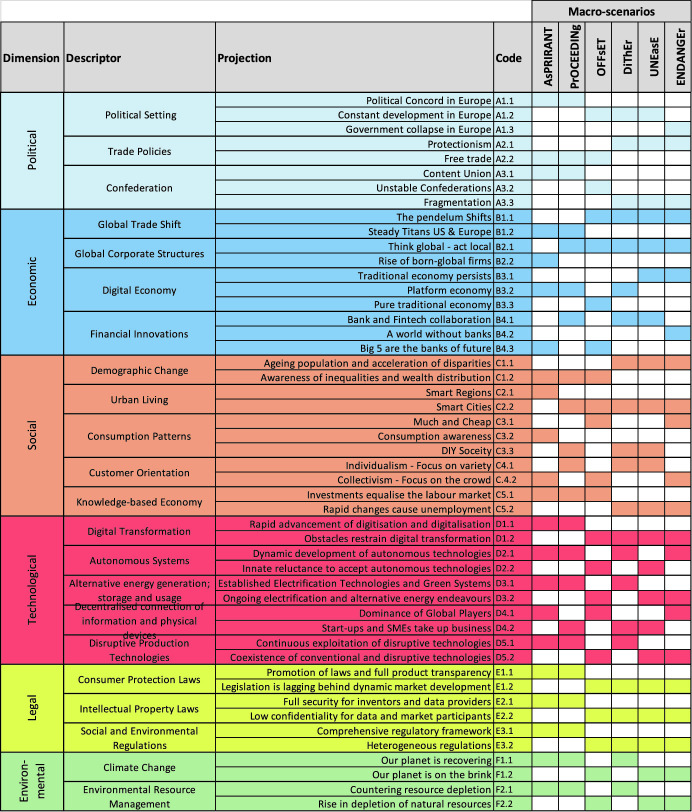
Summary of macro-scenarios

The Consequence Matrix is a table with the decision fields of Table 1 in the lines and the six macro-scenarios in the columns. The inputs to fill in the Consequence Matrix, i.e. to choose the alternative for each macro-scenario, came from three sources. First, the description of the macro-scenarios was used, which is summarized in Table 2. Second, a questionnaire was administered in 2018 and gathered the opinions of 62 experts from process industry, discrete manufacturing, distribution and logistics, ICT industry and academia. For each projection of Table 2, the following question was asked: “Please provide one or more outcomes/changes for your business and supply chain (e.g. SC structure or processes, business model, product portfolio, revenue, staff, IT) that will result from the projection”. Third, a workshop held with 15 experts from industry and 2 from academia at the SCM conference in Portugal in July 2018. During the expert workshops the six scenarios were presented as well as the correspondent product characteristics, and participants were asked to describe the implications of each scenario in the process and supply chain characteristics of their businesses.

The following section describes the supply chains characteristics for each macro-scenario and Table 9, in the conclusions section, presents the overview of the consequence matrix.

## Supply Chains for Macro-Scenarios

This section presents the supply chain characteristics of each macro-scenario. Each table in the sub-sections is one column of the consequence matrix, plus the justifications provided by the experts for the alternative selected in each decision field. The brackets in the tables include the projections of Table 2 that support the supply chain characteristic of each macro-scenario. The full description of the macro-scenarios is available in Sardesai et al. (2020b).

### Supply Chain for Macro-Scenario “aSPIRANT”

In aSPIRANT macro-scenario, economic climate is shaped by the multiplicity and competitive capabilities of born-global firms and platform businesses. According to this scenario, Europe and neighboring regions do not face political upheavals, calamities, or any other political risk factors that affect the demand predictability and interrupt the flow of commerce. Therefore, the product portfolio will be mainly standardized, i.e. supply chains are dominated by mainstream products. Consensual and market-preserving political settings, consolidated by state unions, bring both economic flexibility and market certainty in bargaining process; whereas, soft regulations facilitate liberal trade security, easy access to raw materials and investment finance. In this sense, the predominant supply chain paradigm is “Efficient”, characterized by low supply and demand uncertainty (Lee, 2002; Wagner et al., 2012).

The aSPIRANT scenario is linked to high-tech manufacturing, where servitisation strategy plays an important role in designing the business/operating model of manufacturing processes, which are based on the digital. Technology level in aSPIRANT is increased by: (1) high investments on technology and related processes, (2) training, and R&D; (3) development of new digital technologies and cybersecurity systems; (4) automation of non-value-added activities; and (5) development of technical skills and specialized IT staff. New digital business, data-driven and real- and near-time tracking/traceability technologies contribute to the development of omnichannel sales, demand pooling (leading to raw materials cost savings) and new revenue streams. Thus, this scenario is predominately characterized by global sourcing and distribution and by hyperconnected factories in terms of supply chain configuration. Furthermore, in a scenario with low variety, low supply uncertainty, large production facilities and digital technologies adoption, digital lean manufacturing is the most suitable manufacturing system.

Finally, in terms of sustainability, this scenario is characterized by environmental and social awareness and companies are prone to adopt green and socially responsible closed-loop supply chain strategies. Table 3 presents the summary of the supply chain characteristics for aSPIRANT scenario.

**Table 3:** Supply chain characterization for macro scenario “aSPIRANT”

Decision field	SC characteristics for “aSPIRANT”	Explanation
Product and service	Mainstream products and Servitisation	• Market expansion: geographical (A1.1, A2.2), economic growth in US & Europe (B1.2), new customers from digital business (B1.2, B3.2) • More global competitors (A2.2, A3.1, B2.2, C4.2, D4.1): Small and start-up companies are ‘born global’ (B2.2), Less differentiation, thus more competition (C4.2) • Low variety influenced by: Products for new markets (geographical expansion A1.1, A2.2), Collectivism (C4.2), Consumption awareness (C3.2) • Product portfolio: Standardization (B1.2), More green products (C3.2, F1.1), More data-driven services (B3.2, D5.1)
Supply chain paradigm	Efficient	• Stable demand, due to low variety and standardization (see above) • Stable supply, due to easier access to specific materials and components (A2.2, C2.1, F2.1), many supply sources (A2.2, F2.1), and predictable lead-times (A1.1, A2.2, B1.2, B3.2, D1.1, D2.1)
Sourcing and distribution	Global sourcing Global distribution	• Global sourcing (A2.2, B2.2, D4.1) • Global distribution (A2.2, A3.1, B2.2, D4.1)
Technology level	Digital masters	• High investments (A1.1) on: technology (A2.2, B1.2, D5.1), processes, training (D5.1), and R&D (D1.1) • Digital technologies (B1.2, B3.2) • Automation of non-value-added activities (B1.2, D2.1): high automation in high labour cost country and manual process in low cost country (C5.1), cybersecurity systems (B4.3, E1.1, E2.1), robotic process automation (D1.1, D2.1), electric and hybrid vehicle systems (D3.1) • Technical skills and specialized IT staff required (C2.1, D1.1, D5.1), increased rate of labour force growth (C1.2), increased investment on staff (B1.2), higher skill availability (C5.1)
Supply chain configuration	Hyperconnected factories	• Steady titans US & Europe (B1.2) assure capability for European and US companies to drive global SC. • Global sourcing and distribution enable to digital masters (see above)
Manufacturing systems	Digital lean manufacturing	Low variety, low supply uncertainty, large production facilities, digital technologies adoption (see above)
Sales channel	Omnichannel	Digital transformation (B3.2, D1.1, D5.1) Consumption awareness (C3.2)
Sustainability	Green Social responsible Closed-loop	Green: Environmental awareness (F1.1, F2.1) Social-responsible: Awareness of inequalities and wealth distribution (C1.2) Closed-loop: Digital transformation (B3.2) and environmental awareness (E3.1, F1.1, F2.1)

### Supply Chain for Macro-Scenario “PrOCEEDINg”

The “PrOCEEDINg” macro-scenario is a positive scenario in the sense that most of the trends change in such a way that they help companies with the implementation of innovative SC models, where political and legal situations are stable and new market opportunities are arising from social conditions. This scenario, characterised by political stability and combined with free trade between contended unions, opens up possibilities for wide customisation opportunities. The continuity of power dominance of Europe and the U.S.A., coupled with digital transformations and collaborations between traditional financial establishments and FinTech companies, encourages rapid advancement of digitalisation processes, as well as dynamic development of autonomous technologies. These features, combined with the expected global sourcing and distribution, lead to hyperconnected factories as supply chain configuration. In PrOCEEDINg, start-ups and SMEs will take up business, while global competitors must adapt products to local culture, especially with the advent of a DIY focused society strongly supported by individualism. In this sense, customized product portfolios, servitization, as well as omnichannel and C2C sales channel, as well as digital mass customization as manufacturing systems, are required to succeed on this scenario. This increase in customization leads to an environment characterized by high degrees of demand uncertainty, although with relatively low levels of supply uncertainty—due to the easier access to materials and components, many supply sources and predictable lead-times—requiring companies to adopt strategies that combine characteristics of a lean and agile supply chain strategies, usually know as a leagile strategy (Zimmermann et al., 2020).

With an economy being digitalised in nature and based on growing digital potential, technologies based on the digitalisation concept thrive and receive more R&D investments. However, high automation on developed countries and low automation on underdeveloped countries are expected results, especially when considering the power dominance of the steady titans. The rise of circular economy exposes the need for a closed-loop supply chain, which concerns the circularity in supply chain configurations with restorative and regenerative processes (Batista et al. 2018). This archetype can be integrated with the green supply chain, which relates to scenarios where the decision-making is made based on environmental concerns without much focus on the financial performance (Laari et al., 2016; Melnyk et al., 2010; Salmani et al., 2018). Table 4 presents the summary of the supply chain characteristics for PrOCEEDINg scenario.

**Table 4:** Supply chain characterization for macro scenario “PrOCEEDINg”

Decision field	SC characteristics for “PrOCEEDINg”	Explanation
Product and service	Customized products and servitization	• Market expansion: geographical (A1.1, A2.2), economic growth in US & Europe (B1.2), new customers from digital business (B1.2, B3.2) • Decreased market size and demand of many products due to Do-It-Yourself (DIY) society (C3.3) • More global competitors (A2.2, A3.1); Global competitors adapt their product to the local culture (B2.1); Reduced competition due to needed investments on sustainability (F1.1), • High variety influenced by: products for new markets (geographical expansion A1.1, A2.2), individualism (C4.1), product differentiation for global companies present in local markets (B2.1) • Product portfolio: standardization (B1.2), customization (B3.2, C3.3, C4.1), more green products (F1.1), more data-driven services (B3.2, D5.1), DIY-products (C3.3)
Supply chain paradigm	Leagile	• Unstable demand due to high variety and customization (see above) • Stable supply due to easier access to specific materials and components (A2.2, F2.1), many supply sources (A2.2, F2.1), and predictable lead-times (A1.1, A2.2, B1.2, B3.2, D1.1, D2.1)
Sourcing and distribution	Global sourcing Global distribution	• Global sourcing (A2.2) • Global distribution (A2.2, A3.1)
Technology level	Digital Masters	• High investments on (A1.1): technology (A2.2, B1.2, D5.1), processes, training (D5.1), R&D (D1.1) and education (lifelong learning) • Digital technologies (B1.2, B3.2): Cloud-based software platforms (D2.1); IoT (D2.1); Data Science (D2.1); Communications Infrastructure (D2.1); Cybersecurity systems (E1.1, E2.1) • Automation of non-value-added activities (B1.2, D2.1): high automation in high labour cost country and manual process in low cost country (C5.1); robotic process automation (B1.2, D1.1, D2.1); automated transportation (B1.2, D2.1); self-driving vehicles (D2.1), additive manufacturing of critical parts (D2.1); • Environmentally friendly technologies: Renewable energy technologies; New electrification systems electric and hybrid vehicle systems (D3.1) • Technical skills and specialized IT staff required (D1.1, D5.1), increased rate of labour force growth (C1.2), increased investment on staff (B1.2)
Supply chain configuration	Hyperconnected factories	• Steady titans US and Europe (B1.2) assure capability for European and US companies to drive global SC • Global sourcing and distribution and digital masters (see above)
Manufacturing systems	Digital mass customization	High variety, low supply uncertainty, small and medium production facilities (D4.2), digital technologies adoption (see above)
Sales channel	Omnichannel C2C	Digital transformation (B3.2, D1.1, D5.1) Do-it-yourself society (C3.3)
Sustainability	Green Social-responsible Closed-loop	Green: Environmental awareness (E3.1, F1.1, F2.1) Social-responsible: Awareness of inequalities and wealth distribution (C1.2) Closed-loop: Digital transformation (B3.2) and environmental awareness (E3.1, F1.1, F2.1)

### Supply Chain for Macro-Scenario “OFFsET”

The macro-scenario “oFFsET” can be described in general as a moderated scenario. It is characterised by a partially positive political environment due to open borders and reduced import and export tariffs, which enable the conditions for an agile global sourcing and distribution. From the demand point of view, oFFsET scenario is driven by a moderate market expansion mainly due to a constant development of policies in Europe in a free trade setting, where emerging economies, principally from Asia, open new markets. Due to a moderate market expansion and more global competitors (although with some level of adaption of the products to the local culture) this scenario is characterized by less differentiation, more competition and smaller customer portfolio, leading to the predominance of mainstream products. Due to free trade and an increasing political unrest in countries neighboring Europe, companies need to think glocal in terms of supply and distribution. Additionally, as a result of the low demand uncertainty (due to low variety) and high supply uncertainty (due to resource scarcity), a risk-hedging SC strategy is expected to be predominant, as well modular systems and agile manufacturing systems, with large production facilities.

The existence of ambiguous regulation affects both technology and environment decisions. From the technological point of view, the lack of regulations has a direct impact on digital transformation, impeding a sustained development; only occasionally some technologies are successfully implemented by global companies, which can afford its adoption. In this sense, traditional sales channels are predominant. Ambiguous regulations for the environment, which do not face climate change challenges, combined with increasing global population (mainly living in cities fostering the expansion of urban areas), and thus growing consumerism, are exhausting natural resources. Thus, a resource-efficient social-responsible paradigm tends to be predominant. Table 5 presents the summary of the supply chain characteristics for oFFsET scenario.

**Table 5:** Supply chain characterization for macro scenario “oFFsET”

Decision field	SC characteristics for “oFFsET”	Explanation
Product and service	Mainstream products	• Moderate market expansion: geographical (A1.2, A2.2), new markets in emerging economies (B1.1) • More global competitors (A2.2, C4.2, D4.1): Global competitors adapt their product to the local culture (B2.1), i.e. less differentiation, thus more competition (C4.2), and smaller customer portfolio due to challenges in IP protection (E2.2); Disaggregation of SC (D1.2), i.e. big multinational with advanced IT (D1.2) and small companies will lose IT pace (D1.2) • Low variety influenced by: products in emerging economies (B1.1), collectivism (C4.2), product differentiation for global companies present in local markets (B2.1, D3.2) • Product portfolio: duplication of product portfolios across regions (A1.2), sustainable products for conscious consumers (E3.2)
Supply chain paradigm	Risk-hedging	• Stable demand due to low variety (see above) • Unstable supply due to resource scarcity (F1.2, F2.2)
Sourcing and distribution	Glocal sourcing Glocal distribution	• Glocal sourcing (A2.2, B2.1, D4.1, F1.2, F2.2) • Glocal distribution (A2.2, A3.2, B2.1)
Technology level	Tech conservatives	• Local technological investment (A3.2, B2.1) • Cybersecurity constraints (B3.3, B4.3, D1.2, E2.2) • Automation of non-value-added activities: high automation in high labour cost country and manual process in low cost country (C5.1, D4.2) • Hydrogen power cells and biomass (D3.2) • Lack of specialized staff (D1.2) and increased rate of labour force growth (C1.2)
Supply chain configuration	Modular systems	• Pendulum shifts (B1.1) and much and cheap (C3.1) • Mainstream products and tech conservatives (see above)
Manufacturing systems	Agile manufacturing	Low variety, high supply uncertainty, large production facilities (see above)
Sales channel	Traditional sales channels	Digital impediment (B3.3) Obstacles restrain digital transformation (D1.2)
Sustainability	Resource-efficient Social-responsible	Resource-efficient: resource scarcity (F1.2, F2.2) Social-responsible: Awareness of inequalities and wealth distribution (C1.2)

### Supply Chain for Macro-Scenario “DiThER”

The macro-scenario “DiThER” is a mainly positive scenario as there is an increasing influence of digital transformation, development of autonomous technologies, establishment of electrification technologies and green systems, the continuous exploitation of disruptive technologies and investment in smart cities. Regarding the demand characteristics, the supply chain scenario is based on market contraction, due to protectionism and fragmentation, customization, given that this society is supported on the DIY concept of consumerism, and new markets in emerging countries. Due to the market contraction there will be reduced competition and global competitors will adapt their product to the local culture. Thus, customized products and servitization are required, and, considering the product variety caused by individualism and uncertain demand, there will be a high complexity and mainly small and medium production facilities with flexible manufacturing systems.

Due to protectionism and heterogeneous regulations, there will be moderate supply sources causing uncertain lead-times and a higher suppliers’ risk. An agile supply chain is thus expected to fit with the high demand and supply uncertainty. Regarding the distribution characteristics, the focus will be on local distribution due to protectionism and fragmentation on the political level and C2C will be paramount as sales channel because of individualism, DIY society and digital transformation.

When it comes to the technologies, the focus, especially in the smart cities, will be on environmentally friendly self-driving vehicles, robots and autonomous transport systems. Applications of IoT, data science and communication infrastructure will be widespread, enabling urban manufacturing, mainly due to the dynamic development of autonomous technologies. The environmental awareness will demand green closed-loop supply chain and new environmentally friendly materials will arise given the focus on individualism and DIY. Table 6 presents the summary of the supply chain characteristics for DiThER scenario.

**Table 6:** Supply chain characterization for macro scenario “DiThER”

Decision field	SC characteristics for “DiThER”	Explantion
Product and service	Customized products and servitization	• Market contraction (A1.2, A2.1, A3.3): new markets in emerging economies (B1.1), decreased market size and demand of many products due to DIY society (C3.3) • Reduced competition (A1.2, A2.1); Global competitors adapt their product to the local culture (B2.1), i.e. smaller customer portfolio due to challenges in IP protection (E2.2); Disaggregation of SC (D1.2), i.e. big multinational with advanced IT (D1.2) and small companies will lose IT pace (D1.2) • High variety influenced by: products in emerging economies (B1.1), individualism (C4.1), product differentiation for global companies present in local markets (B2.1) • Product portfolio: overlapping product development activities and portfolios across regions (A1.2), more data-driven services (B3.2, D5.1), DIY-products (C3.3), customization (B3.2, C3.3, C4.1), sustainable products for conscious consumers (E3.2), more green products (F1.1)
Supply chain paradigm	Agile	• Unstable demand due high variety and customization (see above) • Unstable supply due to access to protectionism (A2.1) and heterogeneous regulations (E3.2)
Sourcing and distribution	Local sourcing Glocal distribution	• Local sourcing (A2.1, B2.1) • Glocal distribution (A2.1, B2.1, B3.2)
Technology level	Tech fashionistas	• Investment on: technology (D5.1, F1.1, F2.1) and training (D5.1) • Digital technologies (B3.2): Cybersecurity -constraints (D1.2, E2.2), IoT (D2.1), Data Science (D2.1), Communications Infrastructure (D2.1) • Automation of non-value added activities (D2.1): Autonomous systems (aging population), Robotics, Automated transportation, Self-driving vehicles • Additive Manufacturing of critical parts (D2.1) • Electric and hybrid vehicle systems (D3.1) • Environmentally friendly technologies • Multi-disciplinary staff (technology, market, languages, digital/analytical skills), leadership skills (D5.1), high unemployment rates (C1.1, C2.2, C5.2)
Supply chain configuration	Urban manufacturing	Smart cities (C2.2), think global-act local (B2.1), focus on variety, DIY society (C3.3)
Manufacturing systems	Flexible manufacturing	High variety, small and medium production facilities, autonomous technologies adoption (see above)
Sales channel	C2C (consumer2consumer)	Do-it-yourself society (C3.3) Legislation hinders digital transformation (E1.2, E2.2, E3.2)
Sustainability	Green Closed-loop	Green: Environmental awareness (F1.1, F2.1) Closed-loop: Digital transformation (B3.2) and environmental awareness (F1.1, F2.1)

### Supply Chain for Macro-Scenario “UNEasE”

The scenario “UNEasE” describes an unstable political environment in which companies have to face protectionism, economic uncertainty and alliance collapse. This scenario is also characterised by poor legislations in different fields: from the heterogeneous environmental regulations, which cause a continuous resource depletion, to the laws to protect intellectual property and customer data, which are lagging behind significantly. This creates obstacles for a complete digital transformation of society and companies act mainly in the business to business environment. The traditional economy persists, coexisting with disruptive practices, often used by big players. SMEs and start-ups compete in the local markets where they are able to create a large variety of products to answer to customer individual needs, arising from the DIY trends.

“UNEasE” presents supply chains with customized products, leagile supply chain paradigm, glocal sourcing and local distribution strategies. Moreover, these supply chains are low-tech (tech conservatives) and the supply chain configuration is based on urban manufacturing strategy, aided by flexible manufacturing and traditional sales channels. Customization will become a pivotal instrument to meet customer needs above the barriers created by protectionism and cultural differences, and this will demand more flexibility in the supply chain logistics for delivering the required product mix. Hence, the variety of the demand will spread, and companies will be asked to manage wider product portfolios. From the supply perspective, supply chains will be required to comply with lower costs of sourcing and inbound logistic.

Low levels of new technology adoption and the prevalence of small and medium production facilities in the different manufacturing sectors affect production efficiency, and request additional efforts to minimize resource consumption, in particular concerning water, and carbon emission. This kind of SC features allows to face lockdown similar to the one caused by recent COVID-19 assuring the provision of materials and products at local level. Regarding environmental and social strategies, resource-efficient and humanitarian SC strategies are employed with the aim to quickly react to possible disastrous environments. Table 7 presents the summary of the supply chain characteristics for UNEasE scenario.

**Table 7:** Supply chain characterization for macro scenario “UNEasE”

Decision field	SC characteristics for “UNEasE”	Explanation
Product and service	Customized products	• Market contraction (A1.2, A2.1, A3.3): new markets in emerging economies (B1.1), decreased market size and demand of many products due to DIY society (C3.3) • Reduced competition (A1.2, A2.1); Global competitors adapt their product to the local culture (B2.1), i.e. smaller customer portfolio due to challenges in IP protection (E2.2); Disaggregation of SC (D1.2), i.e. big multinational with advanced IT (D1.2) and small companies will lose IT pace (D1.2) • High variety influenced by: 1) products in emerging economies (B1.1), 2) individualism (C4.1), and 3)product differentiation for global companies present in local markets (B2.1, D3.2) • Product portfolio: duplication of product portfolios across regions (A1.2), customization (C3.3, C4.1), sustainable products for conscious consumers (E3.2), DIY-products (C3.3)
Supply chain paradigm	Agile	• Unstable demand due high variety and customization (see above) • Unstable supply due to resource scarcity (F1.2, F2.2)
Sourcing and distribution	Glocal sourcing Local distribution	• Glocal sourcing (A2.1, B2.1, B3.1, D4.1, F1.2, F2.2) • Local distribution (A2.1, B2.1, B3.1)
Technology level	Tech conservatives	• Low investment leading to less innovation in products and processes (A2.1) • Low level of automation (D2.2) • Cybersecurity constraints (D1.2, E2.2) • Rise of sharing economy (B4.1) • Hydrogen power cells and biomass (D3.2) • Lack of IT Specialized staff (C5.2, D1.2), high unemployment rates (C1.1, C2.2, C5.2)
Supply chain configuration	Urban manufacturing	Smart cities (C2.2), think global-act local (B2.1, D4.2), focus on variety, DIY society (C3.3)
Manufacturing systems	Flexible manufacturing	High variety, small and medium production facilities, autonomous technologies adoption (see above)
Sales channel	Traditional sales channels	Traditional economy persists (B3.1) Obstacles restrain digital transformation (D1.2)
Sustainability	Resource-efficient Humanitarian	Resource-efficient: resource scarcity (F1.2, F2.2) Humanitarian: increased climate change consequences (F1.2)

### Supply Chain for Macro-Scenario “ENDANGEr”

The “ENDANGEr” macro-scenario can be considered as a pessimistic scenario since companies are facing an unstable political environment in Europe, a ‘global trade shift’ from advanced economies towards emerging market economies, as well as protectionism. Moreover, climate change, sanitary crisis, resource scarcity, and the lack of environmental and digital regulations are putting companies to high risks and challenges.

In the “ENDANGEr” scenario, the rise of new business models and digital innovation, the continuous efforts to reduce products’ prices, and the resource scarcity lead to the development of frugal mass products. In fact, companies use frugal mass products to respond to the lack of necessary resources and/or infrastructure and meet their customers’ needs in constrained environments (Mourtzis et al., 2016). One the one hand, markets are composed of different segments that have their own distinct needs and preferences, and companies respond to local needs which are strongly influenced by social networks, leading to relatively low demand uncertainty. On the other hand, supply chains face protectionism (e.g. tariffs on imported goods and import quotas) or lockdowns at global level caused by pandemic diffusion, generating high supply uncertainty and requiring the adoption of risk-hedging supply chain strategies. In terms of manufacturing systems, efficiency and reconfigurability would help companies to face the above-mentioned characteristics.

As protectionism policy restricts the international trade and companies will face barriers and several tariffs, local supply chains will be developed. That means that upstream in the supply chain, companies will source glocally by forming new partnerships and downstream in the supply chain, companies will sell their products locally. Regarding the technological dimension, the political and social instability limits the development of emerging technologies. Due to high costs and risks, the digitalisation is only afforded by big companies. However, that gives the chance to SMEs to develop autonomous technologies and become tech-beginners to face the need to have self-standing production and assure remote working conditions.

In terms of sustainability, companies will be forced to become resource-efficient due to the depletion of resources and they will learn to use the resources in a sustainable manner. The impacts of climate change provoke extreme events and therefore humanitarian supply chains will be prepared to quickly respond to these catastrophes. Table 8 presents the summary of the supply chain characteristics for ENDANGEr scenario.

**Table 8:** Supply chain characterization for macro scenario “ENDANGEr”

Decision field	SC characteristics for “ENDANGEr”	Explanation
Product and service	Frugal products	• Market fragmentation (A1.3, A2.1, A3.3) and new markets in emerging economies (B1.1) • Reduced competition (A1.3, A2.1); Global competitors adapt their product to the local culture (B2.1, D4.1), i.e. less differentiation, thus more competition (C4.2); Smaller customer portfolio due to challenges in IP protection (E2.2); Disaggregation of SC (D1.2), i.e. big multinational with advanced IT (D1.2) and small companies will lose IT pace (D1.2) • Low variety influenced by: products in emerging economies (B1.1), collectivism (C4.2), and product differentiation for global companies present in local markets (B2.1, D3.2, D4.1) Product Portfolio: Duplication of product portfolios across regions (A1.3), sustainable products for conscious consumers (E3.2), mass-market products
Supply chain paradigm	Risk-hedging	• Stable demand due to low variety (see above) • Unstable supply due to resource scarcity (F1.2, F2.2)
Sourcing and distribution	Glocal sourcing Local distribution	• Glocal sourcing (A2.1, B2.1, B3.1, D4.1, F1.2, F2.2) • Local distribution (A2.1, B2.1, B3.1)
Technology level	Tech beginners	• Low investment leading to less innovation in products and processes (A1.3, A2.1) Automation of non-value-added activities (D2.1): Autonomous systems, automated transportation, self-driving vehicles (D2.1) • Cybersecurity constraints (D1.2, E2.2) • Hydrogen power cells and biomass (D3.2) • Lack of IT Specialized staff (C5.2, D1.2), high unemployment rates (C1.1, C2.2, C5.2)
Supply chain configuration	Simple systems	• Pendulum shifts (B1.1) and much and cheap (C3.1) • Frugal mass products and tech beginners (see above)
Manufacturing systems	Efficient and reconfigurable manufacturing	Low variety, high supply uncertainty, large production facilities, autonomous technologies adoption (see above)
Sales channel	Traditional sales channels	Traditional economy persists (B3.1) Obstacles restrain digital transformation (D1.2)
Sustainability	Resource-efficient Humanitarian	Resource-efficient: resource scarcity (F1.2, F2.2) Humanitarian: political instability (A1.3) and increased climate change consequences (F1.2)

## Conclusions

This chapter described six future supply chains scenarios based on eight strategic dimensions: Product and Service, Supply Chain Paradigm, Technology Level, Sourcing and Distribution, Supply Chain Configuration, Manufacturing Systems, Sales Channels, and Sustainability. Table 9 shows the overview of the supply chains’ characterization for each macro-scenario.

The characteristics of the supply chains for each macro-scenario were derived from the macro-scenario projections, complemented by the opinions of experts. The results of this chapter provide the basis for defining the technologies that are needed in each of the future scenarios (Senna et al., 2020), which will then lead to the analysis of Research Priorities and Public Policy Recommendations for the future supply chains in Europe 2030 (Fornasiero et al., 2020).

In this work, a proposal of the supply chain characteristics or features is presented as a support for companies to understand how to link their way of working to the external conditions (political, economic, social, technological, legal and environmental), towards reacting and adapting to them. Given this, it is important to discuss some managerial implications for the definition of a path to innovation starting from the awareness that external and internal conditions are interlinked in the definition of this path.

In particular, macro-scenarios with positive features (such as aSPIRANT and PrOCEEDINg) are characterized by a favourable environment for the technological development and creation of appropriate eco-systems for cross-fertilisation among companies and different sectors. Moreover, in this kind of macro-scenarios, SCs have the right capabilities to respond efficiently to the external environment: companies fully master digitalisation and SCs are hyper-connected, integrated with upstream and downstream and inclusive to valorise humans; in this kind of scenarios it is expected that the research can be easily stimulated with the aim of consolidating the strategies and practices already implemented by networks and springing companies to even higher and better performance as well as doing further important steps to explore highly cutting-edge solutions. Also, implementation of sustainability strategies towards circular economy will be facilitated by the legislation and political conditions.

For the other scenarios, where the external conditions can have negative impact on SC, such as social changes (i.e. increasing aging society bringing difficulties to find young workers), economic restrictions (i.e. protectionism, and large companies monopolies bringing difficulties to find global suppliers and global markets) and legal obstacles (i.e. heterogeneous legislation and lack of consumers’ data protection), SCs innovation advancement is limited by all these impediments. Consequently, the full implementation of adequate SC research policies should be accompanied by actions such as training, creation of adequate infrastructures, definition of adequate finance tools, that will help to increase the readiness to invest in research projects in order to pass from being digital beginner or tech conservative to digital masters. In this case, it is necessary to propose innovation paths to help the supply chains and the companies to increase the technological level of the networks by creating tools and models to face efficiently the challenges and issues of each specific scenario (see Fornasiero et al., 2020).

**Table 9:** Supply chain characterization for macro-scenarios

Decision field	Macro-scenario “aSPIRANT”	Macro-scenario “PrOCEEDINg”	Macro-scenario “oFFsET”	Macro-scenario “DiThER”	Macro-scenario “UNEasE”	Macro-scenario “ENDANGEr”
Product and service	Mainstream products and servitisation	Customised products and servitisation	Mainstream products	Customised products and servitisation	Customised products	Frugal products
Supply chain paradigm	Efficient	Leagile	Risk-hedging	Agile	Agile	Risk-hedging
Technology level	Digital masters	Digital masters	Tech conservatives	Tech fashionistas	Tech conservatives	Tech beginners
Sourcing and distribution	Global sourcing Global distribution	Global sourcing Global distribution	Glocal sourcing Glocal distribution	Local sourcing Glocal distribution	Glocal sourcing Local distribution	Glocal sourcing Local distribution
Supply chain configuration	Hyperconnected factories	Hyperconnected factories	Modular systems	Urban manufacturing	Urban manufacturing	Simple systems
Manufacturing systems	Digital lean manufacturing	Digital mass customisation	Agile manufacturing	Flexible manufacturing	Flexible manufacturing	Efficient and reconfigurable manufacturing
Sales channel	Omnichannel	Omnichannel C2C	Traditional sales channels	C2C	Traditional sales channels	Traditional sales channels
Sustainability	Green Social responsible closed-loop	Green Social responsible closed-loop	Resource-efficient Social responsible	Green Closed-loop	Resource efficient Humanitarian SC	Resource efficient Humanitarian SC
